# The Ratio between Field Attractive and Background Volatiles Encodes Host-Plant Recognition in a Specialist Moth

**DOI:** 10.3389/fpls.2017.02206

**Published:** 2017-12-22

**Authors:** Geir K. Knudsen, Hans R. Norli, Marco Tasin

**Affiliations:** ^1^Division of Biotechnology and Plant Health, Norwegian Institute of Bioeconomy Research, Oslo, Norway; ^2^Integrated Plant Protection, Department of Plant Protection Biology, Swedish University of Agricultural Science, Alnarp, Sweden

**Keywords:** (3E)-4, 8-Dimethyl-1, 3, 7-nonatriene, ultrasonic sprayer, apple fruit moth, *Malus domestica*, *Sorbus aucuparia*, Rosaceae, *Picea abies*, Pinaceae

## Abstract

Volatiles emitted by plants convey an array of information through different trophic levels. Animals such as host-seeking herbivores encounter plumes with filaments from both host and non-host plants. While studies showed a behavioral effect of non-host plants on herbivore host location, less information is available on how a searching insect herbivore perceives and flies upwind to a host-plant odor plume within a background of non-host volatiles. We hypothesized here that herbivorous insects in search of a host-plant can discriminate plumes of host and non-host plants and that the taxonomic relatedness of the non-host have an effect on finding the host. We also predicted that the ratio between certain plant volatiles is cognized as host-plant recognition cue by a receiver herbivorous insect. To verify these hypotheses we measured the wind tunnel response of the moth *Argyresthia conjugella* to the host plant rowan, to non-host plants taxonomically related (Rosaceae, apple and pear) or unrelated to the host (Pinaceae, spruce) and to binary combination of host and non-host plants. Volatiles were collected from all plant combinations and delivered to the test insect via an ultrasonic sprayer as an artificial plume. While the response to the rowan as a plant was not affected by the addition of any of the non-host plants, the attraction to the corresponding sprayed headspace decreased when pear or apple but not spruce were added to rowan. A similar result was measured toward the odor exiting a jar where freshly cut plant material of apple or pear or spruce was intermixed with rowan. Dose-response gas-chromatography coupled to electroantennography revealed the presence of seven field attractive and seven background non-attractive antennally active compounds. Although the abundance of field attractive and of some background volatiles decreased in all dual combinations in comparison with rowan alone, an increased amount of the background compounds (3E)-4,8-Dimethyl-1,3,7-nonatriene ((E)-DMNT) and (Z)-3-hexenyl acetate was found in the rowan-apple and rowan-pear but not in the rowan-spruce headspace. A higher ratio between the abundance of each field attractive component and that of (E)-DMNT and (Z)-3-hexenyl acetate was measured for rowan and rowan-spruce in contrast to rowan-pear and rowan-apple headspaces. Our result suggests that the ratio between field attractive and background antennaly active volatiles encodes host-plant recognition in our study system.

## Introduction

In plant-herbivore associations, volatile plant secondary metabolites are essential cues during host-plant search. Airborne filaments of volatiles from different plants diffuse passively downwind over long ranges and may overlap with each other forming trails with a fluctuating degree of complexity (Myrick et al., 2009). Host-seeking herbivores will encounter plumes with filaments from both host and non-host plants. Although a number of studies have focused on the effect of non-host plants on herbivore host location and oviposition (Zhang and Schlyter, 2004; Ukeh et al., 2010; Jactel et al., 2011) less information is available on how a searching insect herbivore perceives and flies upwind to a host odor plume within a background of non-host volatiles.

Several hypotheses have been proposed to explain the fine-tuned accuracy of host location in an intricate olfactory habitat. Insect host location by taxonomically specific plant compounds is relatively rare (Fraenkel, 1959), although exceptions are reported (Pivnick et al., 1992). More often detection and behavioral responses, even by specialist herbivores, involve ubiquitous plant volatiles (Visser, 1986). In accordance to this concept, host location is proposed to be based on recognition of blend composition via simultaneous detection of blend constituents by co-localized olfactory receptor neurones within the same sensillum (Bruce and Pickett, 2011), as established for sex-pheromones (Baker et al., 1998). In contrast, the interruption of host-location was reported to be mediated by non-host inhibitors (Graves et al., 2008; Jactel et al., 2011).

Volatiles released simultaneously from the same source are transported downwind in pockets of air retaining the blend quality associated with the releasing plant over large distances (Andersson et al., 2011). Although this seems to be sufficient information for plant recognition in a complex habitat (Vickers and Baker, 1994), there are still gaps in our knowledge on how plant recognition is achieved and whether or not host-seeking insects percept and translate into behavioral decisions the information coming from non-host plants (Thomas-Danguin et al., 2014).

In the present study we tested two hypotheses. The first is that an herbivorous insects in search of a host-plant is capable of discriminating plumes of host and non-host plants and that the taxonomic relatedness of the non-host have an effect on finding the host. We hypothesized that in a complex olfactory habitat, a searching insect is capable of locking on to a host plant plume and disregard the co-occurrence of odor filaments containing non-host plant signals.

The second hypothesis is that the ratio between behaviourally active volatiles within a plant odor plume provides the key to discriminate between host and non-host plants. In particular, we predicted that the ratio between kairomone constituents (termed from now on “field attractive”) and background compounds is an explanatory variable for this phenomenon. To test both hypotheses, we examined the wind tunnel response of the moth *Argyresthia conjugella* Zeller to the host plant rowan (*Sorbus aucuparia*), to non-host plants (apple *Malus domestica*, pear *Pyrus communis* and Norway spruce *Picea abies*) and to binary combination of the host and a non-host plant. While apple and pear are both closely related to rowan and to each other (family Rosaceae; Zhang et al., 2017), the Norway spruce represents a rather unrelated and phylogenetically distinct species (family Pinaceae) with distinct composition of released volatiles (Knudsen et al., 2006). Theoretically, spruce specific volatiles could act as non-host interruptants during host-location. While rowan is the preferred host plant by *A. conjugella*, apple is considered as a substitution host in years when rowan does not produce fruits (Satake et al., 2004). Although colonization of pear was reported once, both pear and spruce are not accepted as host-plant by the moth offspring (Bengtsson et al., 2006). For this herbivore a kairomone blend including seven volatiles from rowan was identified and found to be attractive in both rowan forests and apple orchards (Knudsen and Tasin, 2015).

Plant volatiles were collected from every single or plant combination and dispensed in the wind tunnel through an ultrasonic sprayer as an artificially mixed plume. To identify the ability of host odor discrimination in the apple fruit moth the host/non-host mixes were presented heterogeneously, to mimic interspersed odor filaments as found in a natural situation, or homogeneously with a perfect overlap of odor filaments. Chemical and electrophysiological analyses of the sprayed headspace were carried out to screen for ratio between compounds as variables explaining behavioral differences. Established methods such as gas-chromatography coupled to either electroantennography (GC-EAD) or to mass-spectrometry (GC-MS) were selected to these purposes because of their high sensitivity in volatile detection (McCormick et al., 2014). In connection to these properties, a high precision ultrasonic sprayer was chosen as a delivery system of headspace in the wind tunnel to accurately evaluate the behavioral effect of varying ratios of plant volatiles, as recently discussed in another comparable biological system (Salvagnin et al., 2017).

## Materials and Methods

### Insects

Rowan berries infested with last-instar larvae of apple fruit moth were collected in several localities in Southern Norway. The berries were put in paper bags with rolls of corrugated cardboard for pupation. The rolls with diapausing pupae were kept outdoors during winter and transferred to a climatic chamber in spring where they were kept at 4°C until eclosion in Plexiglas cages under an 18L:6D photoperiod, 19–20°C and 60–70% rH. Males and females were kept in the same cages for mating. Behavioral tests were performed in June/July during the apple fruit moth natural flight period. Moths were 4–6 days old when tested in the wind tunnel. All behavioral tests were done 1–6 h into the scotophase.

### Plant Material

**FIGURE 1:**
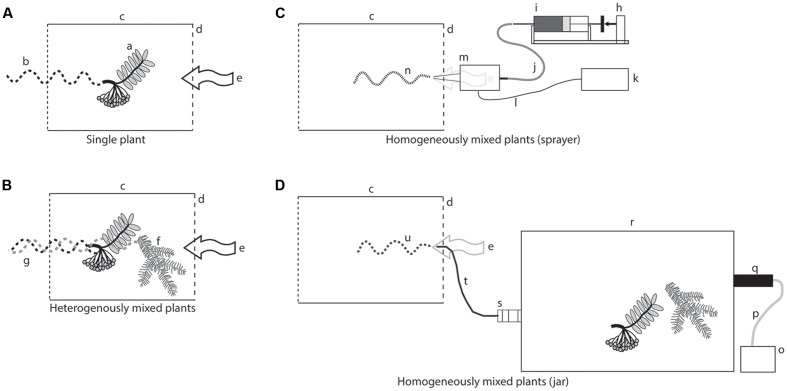
Stimulus delivery devices. **(A)** A plume exiting a glass cylinder with a single plant; a, rowan cluster; b, plume from a rowan cluster; c, glass cylinder; d, upwind side of the wind tunnel; e, air flow entering the cylinder and carrying the volatiles in the wind tunnel. **(B)** A plume exiting a glass cylinder in which two plants of different species were placed; f, spruce twig; g, plume from a spruce twig; the two plumes are freely intermingled. **(C)** An artificial plume generated by an ultrasonic sprayer; h, syringe pump; i, precision syringe delivering an odor collection (from a rowan cluster and a spruce twig); k, function generator; l, electric cable; m, ultrasonic nozzle; n, homogeneous plume generated by the sprayer. **(D)** An artificial plume exiting a jar with the same two plants; o, air pump; p, Teflon tubing for the incoming air; q, charcoal filter; r, glass jar; s, glass fitting to create an artificial turbulence; t, Teflon tubing carrying the plume to the upwind end of the wind tunnel; u, homogeneous plume containing volatiles from the two plants.

Plant material used in the experiments was rowan and apple as these are known to attract apple fruit moths in the field. In addition we tested pear, another rosaceous plant, which is usually not damaged by the apple fruit moth (Bengtsson et al., 2006), and the Norway spruce, which is a non-host plant taxonomically very distant to Rosaceae. Newly cut plant material was used for volatile collections and wind tunnel tests. For rowan, pear and apple both fruits and fresh leaves [<20 cm shoot + 1 cluster or 2–3 (Ø 2–4 cm) fruits] were used. Only growing shoots (<20 cm) were used in the case of spruce. The weight could not be standardized between samples due to differences in growth and fruit size. The weight of the plant material (g) was: 9.2 ± 1.8 (rowan), 21.1 ± 5.7 (apple), 17.8 ± 5.2 (pear), and 6.2 ± 3.1 (spruce). The plant material for the wind tunnel was placed directly into the air stream (see **Figure 1**) without access to water. The same applies for the volatile collections. Volatiles released from the cut is therefore included in the total blend throughout the experiments.

### Headspace Collections

The filters used for collection of plant volatiles were made of 4-mm × 40-mm glass tubes containing 35 mg Super Q (80/100 mesh; Alltech, Deerfield, IL, United States) held between glass wool plugs. Before use, the filters were rinsed sequentially with 6 mL of laboratory grade n-hexane, 6 mL methanol and 6 mL n-hexane again. The plant material was confined in a 2 L glass jar, which was closed with a grounded glass fitting. A charcoal-filtered airstream (SKC Pocket pump 210-1002, SKC Ltd., Eighty Four, PA 15330 United States) was pulled over the plant material from the bottom to the top of the jar and over the Super Q filter. The charcoal filter for incoming air and the collection filter were connected with grounded glass fittings to the jar. Collections were done for 3 h, at 20–22°C from 14:00 to 17:00 h. After sample collection, the traps were desorbed with 0.3 mL of hexane. The outcoming sample (approximately 0.05 mL) was stored at -80°C until use. All glassware was cleaned and heated to 300°C during 8 h before use.

### Chemical Analysis

The measurements were carried out on an Agilent 6890 N gas chromatograph connected to an Agilent 5973 mass spectrometer with an inert ion source. The gas chromatograph was equipped with split/splitless injector operated in splitless mode at 250°C with injection volume 1 μL. The separation column was a fused silica Agilent J&W Scientific DB-WAX 30 m with 0.25 mm internal diameter and 0.25 μm film thickness. A 2.5 m methyl deactivated precolumn (Varian inc., Lake Forest CA, United States) of same internal diameter was connected to the analytical column. The columns were connected by a press fit connector (BGB Analytik, Switzerland). The temperature program was as follows; 40°C held for 2 min, 6.9°C min^-1^ to 160°C, held for 0 min, 21.5°C min^-1^ to 250°C, held for 3.6 min, total time 27.18. The mass spectrometer was operated in scan mode from m/z 40–550, threshold 50 and 2.86 scans/s. Transfer line temperature was set at 280°C, ion source temperature at 230°C and quadrupole temperature at 150°C. The Deconvolution Reporting System (DRS) from Agilent Technologies was used for identification of volatile compounds. The DRS version A.02.00 combines Automatic Mass Spectral Deconvolution and Identification Software (AMDIS) version 2.62, NIST05 database and MSD ChemStation. The AMDIS database contained 1100 volatile compounds, and 180 of them were connected to Kovats retention indexes (Kovats, 1958). To get the same retention times in each setup of samples, the retention time was locked to heptyl acetate at 10.748 min by use of the retention time locking (RTL) software part in ChemStation. Peaks present in the chromatogram, but not found by the DRS software, were identified by manual interpretation by use of NIST05 database. For reliable identification a match factor of ≥70 was employed according to Stein (Stein, 1999).

### Electrophysiology

Antennal recordings were achieved by coupled gas chromatograph – electroantennogram detection, which was performed on an Agilent 6890N GC equipped with an Agilent J & W Scientific DB-Wax capillary column (length 30 m, inner diameter 0.25 mm, film thickness 0.25 μm), with a split/splitless injector and connected to an electroantennogram device (Syntech, Hilversum, Netherlands). The temperature program was the same as for the GC-MS, described above. The effluent from the GC column was split at a ratio of 1:1 between the flame ionisation detector (FID) and a pair of *A. conjugella* antennae that had been removed from the moth by use of micro-scissors and forceps and then connected to the EAD. The effluent led to the EAD was delivered through a heated transfer line (Syntech) into a stream of humidified air in a glass tube (diameter 8 mm, length 12 cm) to the antennae. The excised antennae were mounted in a holder (Syntech) between two silver electrodes, and electrically conductive gel (Parker, Fairfield, NJ, United States) was used to facilitate the contact between the electrodes and the antennae. The tips of the antennae were carefully inserted in a thin layer of the gel that had been applied to the surface of the electrodes. The antennal signal and the FID signal were amplified and recorded simultaneously using Syntech software. Field attractive volatiles in accordance to Knudsen and Tasin (2015) along with the major compounds identified in the headspace of rowan, pear, apple, and spruce were submitted to a natural logarithmic dose-response GC-EAD (*N* = 3). Neat compounds were dissolved in hexane at concentration of 2, 6, 20, 60, and 200 ng per microliter which translates to 1, 3, 10, 30, and 100 ng delivered at the antennae after the 1:1 split. The injected volume was 1 microliter. Groups of compounds were mixed together based on their retention time on the DB-Wax column to achieve good separation between individual peaks.

### Wind Tunnel Hardware

The hardware, technical information and detailed construction of the wind tunnel are described by Aak et al. (2010). Air was blown into the tunnel (67 cm × 88 cm × 200 cm) through a dust filter and 24 active charcoal filters. Between these filters and the flight arena there was a 30 cm section with a perforated metal grid on each side to reduce turbulence. The air from the tunnel was exhausted through a similar filter system as described above before it was released back into the room. Wind speed was set at 30 cm s^-1^ for the experiments and the temperature, humidity and white light intensity was 19–20°C, 55–65% and <1 lux, respectively.

### Odor Delivery

Live plant material for the single plants and for the heterogeneously mixed plumes was carefully fitted inside a glass cylinder with open ends (**Figures 1A,B**; Ø 100 mm; length 120 mm). The cylinder was placed horizontally in the center of the upwind end of the tunnel. A metal grid (1 mm mesh) was covering on the downwind side of the cylinder. The cylinder and metal mesh was used in all wind tunnel experiments, to give the same visual cue for the insects and to serve as a landing platform for the moths.

A homogenous mixed plume was achieved with releasing headspace collections from an ultrasonic sprayer with a conical nozzle. A broadband ultrasonic generator working at 120 KHz (NZL 120, SonoTek, New York, United States) vibrates the nozzle to release the headspace collection as a fine mist (droplet size 18 microns SonoTek 2002). The nozzle had an inserted microbore to restrict the liquid flow to facilitate a release rate of 10 μL min^-1^. Constant liquid flow through the nozzle was achieved using a syringe pump (CMA 102, CMA/Microdialysis AB, Solna, Sweden) connected via a FEP tube (0.12 mm ID, CMA/Microdialysis AB, Solna, Sweden). The sprayer was hidden behind the perforated metal grid and the tip of the nozzle was fit into one enlarged pore of this metal grid, 30 cm from the ground, in the center of the upwind end of the wind tunnel. The tip of the nozzle protruded 1 cm into the tunnel and was covered by the same glass cylinder and metal mesh as described above. Through a preliminary test using sprayed headspace collected from rowan, we observed an optimal response of the moths at a release of 10 μl min^-1^. Headspace samples were diluted with pure ethanol to give a sample volume of 1800 μl. This amount corresponds to the 3 h collection time at 10 μl min^-1^. The delivery circuit (including the nozzle) was cleaned for 10 min with pure ethanol between treatments (**Figure 1C**).

To homogenize the odors from the mixed plant material without spraying the headspace with the ethanol solvent, freshly cut plants were put in a 2 L volatile collection jar, and air was pushed through to the wind tunnel. To ensure homogenous mixing of the released odors further, a small glass connection with a glass wool plug was inserted between the jar and the exhaust tube to increase turbulence. Clean laboratory air was vented into the jar at 1.2 L min^-1^ (VA-flow meter, Sho-Rate 1355, Brooks, Holland) and released into the center of the wind tunnel through a glass tube (Ø 3 mm × 5 cm). The same glass cylinder and metal mesh, as described above, covered the exhaust tube (**Figure 1D**).

### Wind Tunnel Protocol

Three females were put into glass tubes (2.5 cm × 15 cm) covered with gauze on one end and plastic cap at the other. Insects were introduced into the tunnel by positioning the glass tube onto a holder, 180 cm downwind from the source and 30 cm from the ground, and the cap on the upwind end was removed. Insects were given 5 min to respond and the following types of behavior were recorded; (1) oriented flight; upwind over 40 cm toward the source and (2) approaching the source at the glass cylinder after 180 cm of upwind-oriented flight in the center of the wind tunnel. Three to four tubes with insects were tested per odor source per day depending on availability. Each odor source was tested on a minimum of three different days and up to six treatments, in random order as tested each day. Insects were used only once.

### Treatments

An overview of the wind tunnel treatments is presented in **Table 1**. First we established the attractive potential of each plant species in the wind tunnel (**Figure 1A**). To achieve a heterogeneously mixed plume, the main host rowan was tested in combination with each of the other three plants to determine if host-searching females could distinguish the host plant signal in the heterogeneously mixed plume (**Figure 1B**).

**Table 1:** Overview of treatments.

Single plant	Heterogeneously mixed	Homogenously mixed	
		Sprayed headspace collection	Headspace from jar
Rowan	–	Rowan	Rowan
Pear	rowan + pear	rowan + pear	rowan + pear
Apple	rowan + apple	rowan + apple	rowan + apple
Spruce	rowan + spruce	rowan + spruce	rowan + spruce

The attraction to homogeneously mixed plumes was measured through two types of wind tunnel bioassays, using either a plume from the piezoelectric sprayer (**Figure 1C**) or from a glass jar (**Figure 1D**). Simultaneous volatile collection of rowan and the other three plants (pear, apple, and spruce) achieved the headspace for the piezoelectric sprayer. For comparison the attraction of rowan headspace collection was established. With the aim to exclude any possible interference of the ethanol solvent on the behavior, the attraction was also measured toward the headspace from plant material combinations exhausted from a glass jar. In order to properly mix the plumes, the odor exiting the jar was forced through a glass fitting.

### Rationale and Statistics

The software R 3.1.0 was used for statistical analyses. The number of females that exhibited upwind flight or approached the source in the flight tunnel was analyzed by a generalised linear model (GLM) with a binomial distribution and a log it link. A χ^2^ was used to assess for significance of the factor. Levels within the independent variable were separated through 1 × 1 comparisons by a binomial test.

Gas-chromatography coupled to either electroantennography (GC-EAD) dose response data are presented as natural logarithmic regression. Compounds were ranked in a decreasing order according to the intercept at the *Y*-axis.

In order to visually highlight variations in the proportion of antennaly active volatiles due to the mixing process, a dual plant headspace was overlapped with rowan alone through a radar graph. Differences in headspace composition were then plotted in a box plot as ratio to major background components. Additional graphic representation in a multivariate space were done using non-metric multi-dimensional scaling (NMDS) (R package “vegan,” data not shown). Once major compounds were highlighted, the contribution of each compound in the determination of the difference between plant headspaces was evaluated through an analysis of similarities (ANOSIM) (Piper et al., 2017). When comparing the sprayed headspace of attractive plants (rowan and rowan+spruce) with those with a reduced attraction (rowan+pear and rowan+apple), we grouped the antennaly active volatiles into field and background. In a first ANOSIM the entire range of both categories of compounds was used and a ratio between the two was calculated. In the following ANOSIM, while all field attractive compounds were considered, only those with an increased release in the Rosaceae mix, i.e., (3E)-4,8-dimethyl-1,3,7-nonatriene [(E)-DMNT] and (Z)-3-hexenyl acetate, were taken into consideration among the background ones.

## Results

### Attraction to Single Plants

**FIGURE 2:**
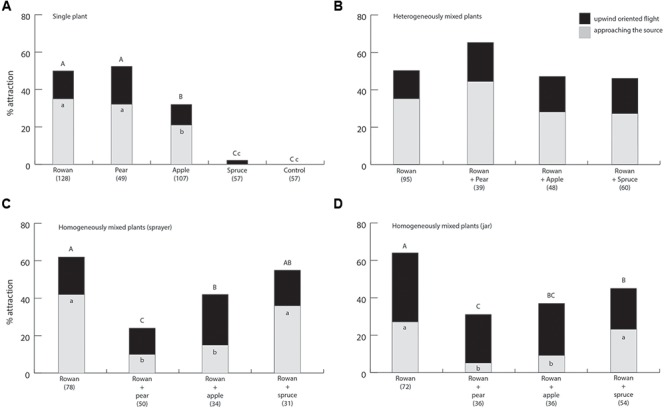
Percentage oriented flights and landings at the source of female apple fruit moths to **(A)** single plant sources, **(B)** heterogeneously mixed plant plumes, **(C)** homogeneously mixed plumes from sprayed headspace and **(D)** homogeneously mixed plumes from plant material in a jar, in a wind tunnel. Numbers in parentheses are total number of moths tested. Within each figure, different capital and lower case letters indicate significant differences between oriented flights and landings at source, respectively.

There were significant differences, in both oriented flight and landing at the source between single plant material treatments. Attraction and approaching the source to rowan and pear were higher than to apple. Apple triggered a higher response in comparison to spruce and control (**Figure 2A**) (df = 4, 398; *P* < 0.001 for both oriented flight and landing).

### Attraction to Heterogeneously Mixed Plumes

No significant differences were found in either oriented flight or landing when a rowan cluster was presented together with each of the three non-host plants (**Figure 2B**; df = 3, 242; *P* = 0.227 and 0.260 for oriented flight and landing, respectively).

### Attraction to Homogeneously Mixed Plumes

Significant differences in attraction were found for both oriented flights and approaching the source when spraying the mixed headspace collections as an artificially mixed plume (**Figure 2C**; df = 4, 193; *P* < 0.001 and *P* = 0.014 for oriented flight and landing, respectively). Oriented flights to the rowan-pear mix were significantly lower than to the rowan headspace, to the rowan-spruce mix and to the rowan-apple mix. Oriented flights to the headspace from the rowan-apple mix were not significantly different from the rowan-spruce headspace, but they differ from rowan and from rowan-pear. Approaching the source to the rowan-apple mix and to the rowan-pear mix was significantly lower than to the rowan headspace and to the rowan-spruce mix. There were significant differences in attraction for both oriented flights and landing at the source when releasing headspace from a jar. Attraction to the homogenous mixed blend of rosaceous plant material was significantly lower than to rowan alone. The number of moths approaching the source significantly differed among mix of rosaceous plants and rowan containing headspaces (**Figure 2D**; df = 4, 198; *P* < 0.001 and 0.033 for oriented flight and landing, respectively).

### Gas-Chromatography Coupled to Either Electroantennography (GC-EAD)

**FIGURE 3:**
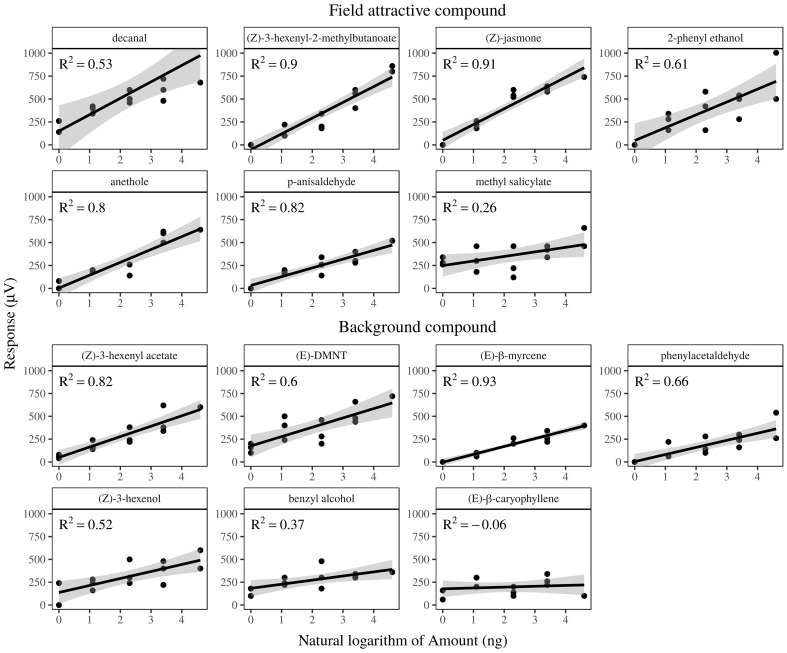
Regression fit of gas-chromatographic electrophysiological dose-response of *Argyresthia conjugella* females to plant volatiles. Dose 1–5 correspond to 1, 3, 10, 30, and 100 ng delivered. Responses on the *Y*-axis are in μV. Compounds are ordered according to their behavioral activity (field or non-field active). The following compounds did not elicit any detectable response with the tested dose: α- and β-pinene, 3-methyl-3-buten-1-ol, 3-methyl-2-buten-1-ol, 3-methyl-1-butanol, α-cubebene and eucalyptol. Germacrene D and α-farnesene were not tested due to their fast degradation when exposed to field conditions.

The antennae of the apple fruit moth responded to 14 out of the 21 tested volatiles (**Figure 3**). All compounds already reported as field attractive by previous studies triggered a significant dose response in this study. While methyl salicylate exhibited the highest value, the ester Z-(3)-hexenyl-2-methylbutanoate showed the lower *Y*-axis intercepting value. Antennae responded consistently to the entire range of doses of each compound, from 1 to 100 ng.

An additional seven compounds elicited an electrophysiological response, although not reported as field attractive in the literature. Among them, (E)-β-caryophyllene did not show a clear dose response pattern, while (E)-DMNT, benzyl alcohol and (Z)-3-hexenol had the highest intercept value. The compound α- and β-pinene, 3-methyl-3-buten-1-ol, 3-methyl-2-buten-1-ol, 3-methyl-1-butanol, α-cubebene and eucalyptol did not elicit antennal responses. (E)-β-myrcene elicited a significant electrophysiological response in the moth antennae and was emitted in large amounts exclusively by spruce. Because its content in the rowan -spruce combination did not affect attraction, we did not take this compound into consideration when analyzing the effect of ratios.

### Chemical Analysis

**FIGURE 4:**
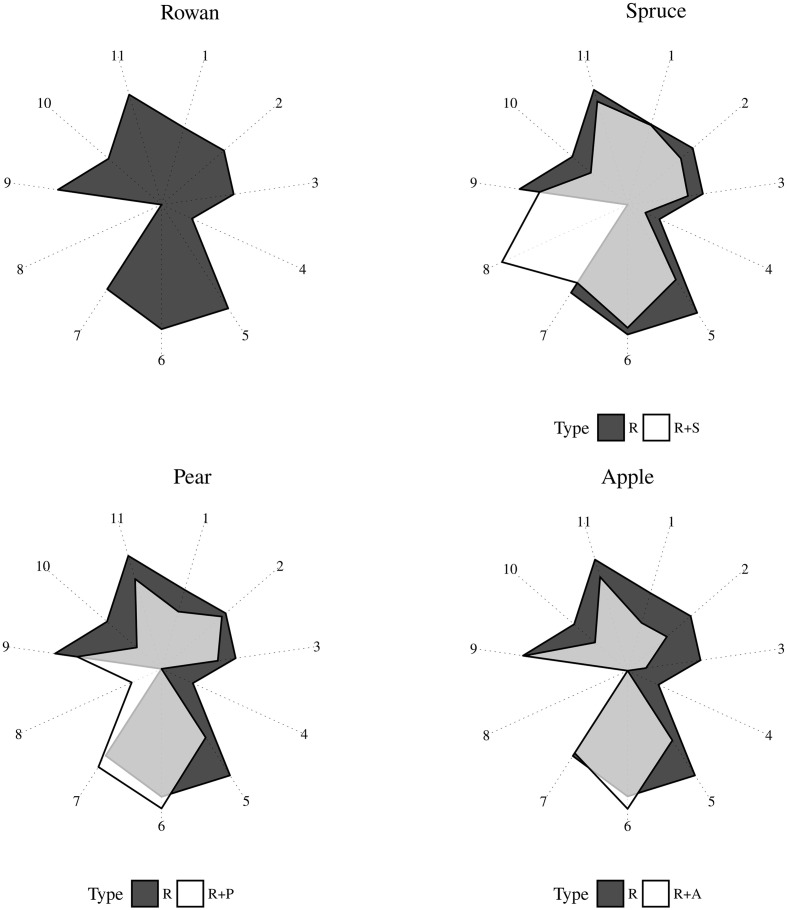
Radar plot representing the proportion of GC-EAD active volatiles in plant headspace collections sprayed in the wind tunnel. R (rowan), S (spruce), P (pear), A (apple). Compounds are coded as follow: (1) decanal, (2) Z-(3)-hexen-2-methylbutanoate, (3) 2-phenyl ethanol, (4) p-anisaldehyde, (5) methyl salicylate, (6) Z-(3)-hexenyl acetate, (7) (E)-DMNT, (8) (E)-β-myrcene, (9) Z-(3)-hexenol, (10) benzyl alcohol, (11) (E)-β-caryophyllene.

The proportion of compounds in the different plant headspace collections is presented in **Figure 4**. (Z)-jasmone, phenylacetaldehyde, and anethole were discarded from this representation because they were detected in less than two analyses across the entire chemical dataset. The compound (E,E)-α-farnesene was found to be EAD active in a previous study whilst germacrene D was not (Bengtsson et al., 2006). While germacrene D and (E,E)-α-farnesene were present in a large number of the collections in this study, we deliberately excluded them from any further antennal and behavioral assay due to their unavailability as high purity synthetic compounds.

When spruce was added to rowan, an increase in the proportion of (E)-β-myrcene occurred. At the same time, a decrease in all the other compounds but not decanal could be observed. The addition of apple or pear to rowan resulted in a decrease of five field attractive compounds. While the reduction in the proportion of methyl salicylate was comparable across all three plant combinations, a much stronger decrease of p-anisaldehyde occurred in the two combinations with rose plants than in rowan plus spruce. In rowan plus pear a decrease of (Z)-3-hexenyl-2-methylbutanoate and 2-phenyl ethanol was measured in comparison with rowan plus apple. While the proportion of (E)-DMNT increased in rowan plus pear, the proportion of (Z)-3-hexenyl acetate increased when either apple or pear were added to rowan. (E)-β-myrcene increased in rowan plus pear, but was not found in rowan plus apple. A reduction in the proportion of (Z)-3-hexenol and benzyl alcohol was observed across all plant combinations. A higher decrease of (E)-β-caryophyllene occurred in rowan plus pear than in the other two plant mixes.

**FIGURE 5:**
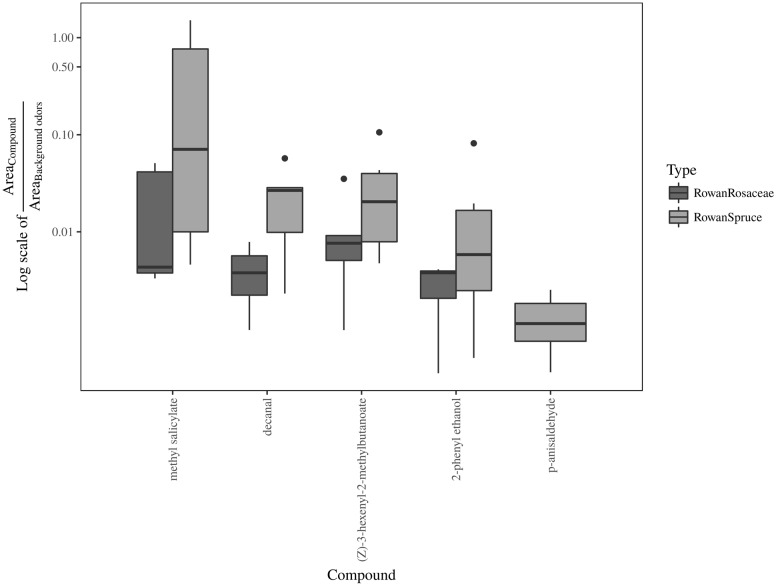
Boxplot representing the ratio between field attractive and background GC-EAD active volatiles in sprayed plant headspace with unchanged (rowan and rowan+spruce) or reduced (rowan+pear and rowan+apple) attraction in comparison with authentic plants.

**FIGURE 6:**
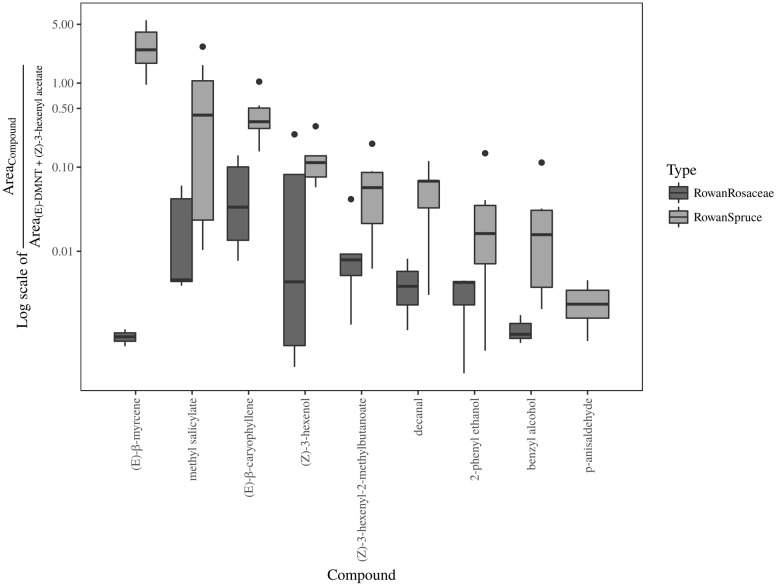
Boxplot representing the ratio between field attractive and two background [(E)-DMNT + (Z)-3-hexenyl acetate] GC-EAD active volatiles in sprayed plant headspace with unchanged (rowan and rowan+spruce) or reduced (rowan+pear and rowan+apple) attraction in comparison with authentic plants.

Result from the ANOSIM showed a non significant difference between rowan and rowan+spruce vs. rowan+Rosaceae when the entire range of field and background GC-EAD active compounds are included [**Figure 5**; *P* = 0.49, *R* = -0.03; contribution to the variation: methyl salicylate 68%, decanal 14%, (Z)-3-hexenyl-2-methylbutanoate 13%, 2-phenyl ethanol 5%]. However, when only those increasing in the rowan+Rosaceae mixes ((E)-DMNT and (Z)-3-hexenyl acetate) were included among the background, the ANOSIM became significant [**Figure 6**; *P* = 0.01, *R* = 0.57; contribution to the variation: methyl salicylate 29%, decanal 2%, (Z)-3-hexenyl-2-methylbutanoate 2%, 2-phenyl ethanol 1%, (E)-β-myrcene 35%, (Z)-3-hexenol 12% and (E)-β-caryophyllene 17%].

## Discussion

In this study we provide evidence that female of a specialist herbivore interprets the volatile language of plants via ratio between plant volatile components. Although already hypothesized by other authors, we provide here a direct and precise measurement of such ratios through chemical analysis coupled to a high precision delivery sprayer.

Alteration of the authentic host plant signal via change in blend ratio underlies moth attraction in the present study. In particular, a strong reduction in ratios between field attractive and background components reflects the observed behavioral variation.

The abundance of field attractive (decanal, (Z)-3-hexenyl-2-methylbutanoate, 2-phenyl ethanol, p-anisaldehyde and methyl salicylate) and of some background volatiles [(Z)-3-hexenol, benzyl alcohol and (E)-β-caryophyllene] decreased in all dual combinations in comparison with rowan alone. An increased proportion of the background compound [(Z)-3-hexenyl acetate] was found in the rowan-apple and rowan-pear combinations while (E)-DMNT increased in the rowan-pear combination. There was no increase of (Z)-3-hexenyl acetate or (E)-DMNT in the rowan-spruce headspace. A higher ratio between the abundance of each field attractive component and that of the sum of (E)-DMNT and (Z)-3-hexenyl acetate was measured for rowan and rowan-spruce in contrast to rowan-pear and rowan-apple headspaces. According to the chemical analysis, we argue that in the sprayed plumes of rowan with pear and rowan with apple a suboptimal proportion between the mentioned components may contribute to the observed decay in source-contact.

The behavioral effect of (E)-DMNT on insects is under investigation, since its role at an ecological level spans over several trophic layers. (E)-DMNT is involved in attraction of herbivores as a constitutive homoterpene of a number of plants as well as in the recruitment of natural enemies as an induced plant volatile (Azuma et al., 1997; James, 2003). It appears, however, that (E)-DMNT could decrease host-plant location in the moth *Spodoptera littoralis* (Hatano et al., 2015) and *Paralobesia viteana* (Cha et al., 2011) but not in *Lobesia botrana* (Tasin et al., 2006) and *Cydia pomonella* (Knight et al., 2011). In our study, a higher release of this homoterpene from the sprayer was correlated with a reduced upwind flight and landing of our tested species. In nature plants releasing a higher amount of (E)-DMNT might be already under herbivory and thus avoided by searching adult moths.

Because the addition of pear had a stronger inhibitory effect in comparison to apple, it appears that a lower amount of (Z)-3-hexenyl acetate in apple accounted for a lower distortion than the rather large decrease in decanal, (Z)-3-hexenyl-2-methylbutanoate and 2-phenyl ethanol. This is confirmed by Knudsen and Tasin (2015), who found that the wind tunnel attraction of a blend of all seven identified field attractive compounds was largely enhanced in the presence of an apple branch as a background. From this experiment it seems that the moth requires the co-occurrence of background volatiles to place the attractive signal in a real context and reach the source. Results from our experiments suggest that the ratio between field attractive and background volatiles embedded within a plant odor is perceived by a flying moth as a host-plant recognition cue. However, only a subset of these vegetative compounds are enough to elicit attraction to a synthetic blend in a field setting, where a background of volatiles is already present.

The influence of blend composition, both as number of components and their relative ratio, has been the subject of several studies (Bruce and Pickett, 2011).

For example, Tasin et al. (2006) and Salvagnin et al. (2017) found that non-host blend proportion of three attractive grape host compounds significantly reduced upwind orientation in *L. botrana*. In an 11-component blend identified from *a* host plant, however, there was a degree of plasticity in the different compositions eliciting behavioral response (Tasin et al., 2010). For polyphagous herbivores such as *L. botrana*, a large plasticity in the olfactory response is expected to accommodate the large range of possible hosts. Najar-Rodriguez et al. (2010) reported a behavioral plasticity and a corresponding odor-evoked neural activity patterns in the antennal lobe to a 100-fold change of the main component in a synthetic plant volatile blend attracting females of the oligophagous moth *Grapholita molesta*. They argue that this plasticity ensures host location when volatile blend change with seasonal advancement. Nonetheless, the effect of background compounds was not assessed in those studies.

In our study, it is intriguing that even with the complete host signal present, alteration of blend configuration strongly diminishes plant-recognition efficiency. When headspace collections from two plants were presented as a unique and artificially mixed plume, a similar landing frequency to rowan was only achieved when the host was mixed with the taxonomically and chemically distinct non-host plant, spruce. The reduced source-contact to homogenous plumes of rosaceous plants can be explained by an altered ratio in the mix, resulting from an additive release of behaviourally active substances from both rosaceous plants. When presented as heterogeneous plume, which reflects a more natural situation, females could disentangle behaviourally active compounds from rowan as well as from apple and pear, as the released volatiles travel downwind in distinct filaments.

The apple fruit moth is a monophagous herbivore and a lower degree of behavioral plasticity compared to polyphagous herbivores is expected. Nonetheless, a proportion of moths still flew upwind and landed at the artificially sprayed plume, indicating that the olfactory system in *A. conjugella* accommodate for some degree of plasticity. Such a plasticity may be essential when the moth shifts from rowan to apple in years with a low rowan berry fruit set. More generally, the capability of responding to suboptimal ratio could either accommodate for a variable host plant signal (Cha et al., 2011) and allow the colonization of additional species, which may be or not incorporated into the host range according to ecological fitting (Agosta, 2006).

Because the calculated ratio between components in a plant headspace may also vary according to the efficiency of the adsorbent and the following solvent washing (Agelopoulos and Pickett, 1998), our result will benefit of a confirmation through experiments with synthetics delivered at a different ratio in both a laboratory and field setting. The chemical selectivity of the volatile collection method and the 3 h collection time may result in a different blend ratio compared to the natural release from the plant used as such, in the wind tunnel (Beck et al., 2012). For example, due to the use of hexane as a solvent, a change in the proportion between polar and non-polar compounds may occur during the desorption process. As a consequence, a discrepancy between the original plant odor and the sprayed headspace cannot be excluded in our study. In addition to this, we cannot preclude the possibility that compounds excluded from the behavioral and antennal assay because of their very limited detection in the data set [(Z)-jasmone, phenylacetaldehyde, anethole] or their low purity as synthetics (germacrene D and α-farnesene) may contribute to host-plant recognition. Several authors point out that after cutting, the plant death commences and may alter the release even over short periods (Rodriguez-Saona et al., 2005). However, in most cases, the wounded plant was found to be at least as attractive as the undamaged control plant. Also, due to a divergent shape of the respective plumes, the concentration of the sprayed headspace may differ from that of the live plant material. Nevertheless, in our conditions, a comparable attraction to rowan live plant material and rowan headspace indicates a comparable release of volatiles. In contrast, a lower response was measured to the plant material placed in a jar. This may be ascribed to a significant change in plume structure in comparison to the heterogeneously mixed plumes.

The antennae of the apple fruit moth showed a selective response, with five of the field attractive volatiles triggering the highest slope value [decanal, 2-phenyl ethanol, anethole, (Z)-jasmone and (Z)-3-hexenyl-2-methylbutanoate] and the most common spruce volatiles, α- and β-pinene, being not detected. In insects, it is, however, not clear whether or not the slope of the electrophysiological response can be correlated to the behavioral output, because additional factors such as modulation of the signal at the brain level can affect the signal processing. We employed here the electrophysiological activity as criteria to restrict the range of compounds considered to play a major role in host-recognition, as done in a number of other studies. Although we are confident that through our dose-response analysis we highlighted the majority of antennaly active compounds in the headspace (Bengtsson et al., 2006), compounds active on another type of insect receptors may contribute to behavioral responses (Silbering and Benton, 2010).

Although lower source-contact was obtained with odor exiting the glass-jar compared to the authentic rowan or its sprayed headspace, results from this assay confirmed once more the neutral influence of spruce odor on source contact to rowan. The behavioral negative effect of pear was also confirmed, while a more accentuated effect of apple emerged. Through this experiment we could rule out any possible unknown interference of the solvent to dilute the sprayed headspace.

In contrast to that, we need to remark that our considerations are extrapolated from a laboratory experiment, with tested insects flying in a plant odor plume within a charcoal purified air background. It remains to be known how the observed behavior will change in a natural setting, where a number of additional odor cues may intermingle in complex plumes to shape the behavior of the herbivore (Tasin et al., 2012; Cai et al., 2017).

Our data provides new knowledge on how a specialist searching herbivore deciphers the language embedded in a plant volatile blend. Although additional investigations involving generalist herbivores may further tailor our concept in a wider ecological context, we reflect that the proposed mechanism of host-plant recognition contributes to the advancement of knowledge on insect-plant interactions, including host-plant recognition and the formation of new herbivore-plant associations.

## Author Contributions

GK wrote the grant, planned the study, performed the experiments and wrote the paper. HN performed chemical analyses. MT planned the study, performed the experiments and the statistics, wrote the paper.

## Conflict of Interest Statement

The authors declare that the research was conducted in the absence of any commercial or financial relationships that could be construed as a potential conflict of interest.
